# Translation Complex Profile Sequencing Allows Discrimination of Leaky Scanning and Reinitiation in Upstream Open Reading Frame-controlled Translation

**DOI:** 10.1016/j.jmb.2024.168850

**Published:** 2024-10-30

**Authors:** Dmitri E. Andreev, Jack A. S. Tierney, Pavel V. Baranov

**Affiliations:** 1 Shemyakin-Ovchinnikov Institute of Bioorganic Chemistry, RAS, 117997 Moscow, Russia; 2 Belozersky Institute of Physico-Chemical Biology, Lomonosov Moscow State University, 119992 Moscow, Russia; 3 School of Biochemistry and Cell Biology, University College Cork, Cork T12 K8AF, Ireland; 4 SFI Centre for Research Training in Genomics Data Science, University College Cork, Cork T12 K8AF, Ireland

**Keywords:** TCP-seq, ribo-seq, uORF, leaky scanning, reinitiation

## Abstract

Upstream open reading frames (uORFs) are a class of translated regions (translons) in mRNA 5′ leaders. uORFs are believed to be pervasive regulators of the translation of mammalian mRNAs. Some uORFs are highly repressive but others have little or no impact on downstream mRNA translation either due to inefficient recognition of their start codon(s) or/and due to efficient reinitiation after uORF translation. While experiments with uORF reporter constructs proved to be instrumental in the investigation of uORF mediated mechanisms of translation control, they can have serious limitations as manipulations with uORF sequences can yield various artefacts. Here we propose a general approach for using translation complex profiling (TCP-seq) data for exploring uORF regulatory characteristics. Using several examples, we show how TCP-seq could be used to estimate both repressiveness and modes of action of individual uORFs. We demonstrate how this approach could be used to assess the mechanisms of uORF-mediated translation control in the mRNA of several human genes, including *EIF5, IFRD1, MDM2, MIEF1, PPP1R15B, TAF7*, and *UCP2*.

## Introduction

Translation initiation in eukaryotes begins with 43S ribosome preinitiation complex (PIC) loading onto the 5′ end of mRNA. This is followed by scanning – a stepwise 5′–3′ PIC movement, until a start codon, usually AUG, is recognized through successful establishment of codon-anticodon interaction in the ribosome P-site.^1–5^ The scanning mode of translation initiation implies that the 5′ proximal AUG codon of mRNA will most likely be the initiation site. Counterintuitively, for ~50% of mammalian mRNAs, the start codon of the protein-coding translon (CDS) is preceded by at least one upstream AUG-initiated uORF.^6–9^ From the scanning mechanism perspective, any uORF can be an obstacle for scanning ribosomes, therefore uORFs are considered to be the most prevalent elements that repress mRNA translation.^10–18^ Ribosome profiling, a technique which can detect mRNA footprints of translating 80S ribosomes at genome-wide level,^19^ allows the identification of thousands of mammalian translated uORFs, including those that use non-AUG initiation codons.^20^

In theory, the inhibitory potential of a uORF depends on the efficiency of initiation at its start codon and the ability of post-terminating ribosomes to resume translation downstream. An inefficient uORF start codon allows leaky scanning and subsequent translation of CDS.^2,11^ In addition, the translation of a uORF can be followed by the reinitiation of translation when the post-terminating 40S subunit gains initiation factors and resumes the scanning process. It is believed that the ribosomes’ dwell time on a uORF negatively correlates with reinitiation efficiency due to the loss of initiation factors bound to the 80S ribosome.^11,21,22^ Substantial leaky scanning and reinitiation at uORFs can explain how 5′ leaders with dozens of uORFs are able to support CDS translation.

**Figure 1:**
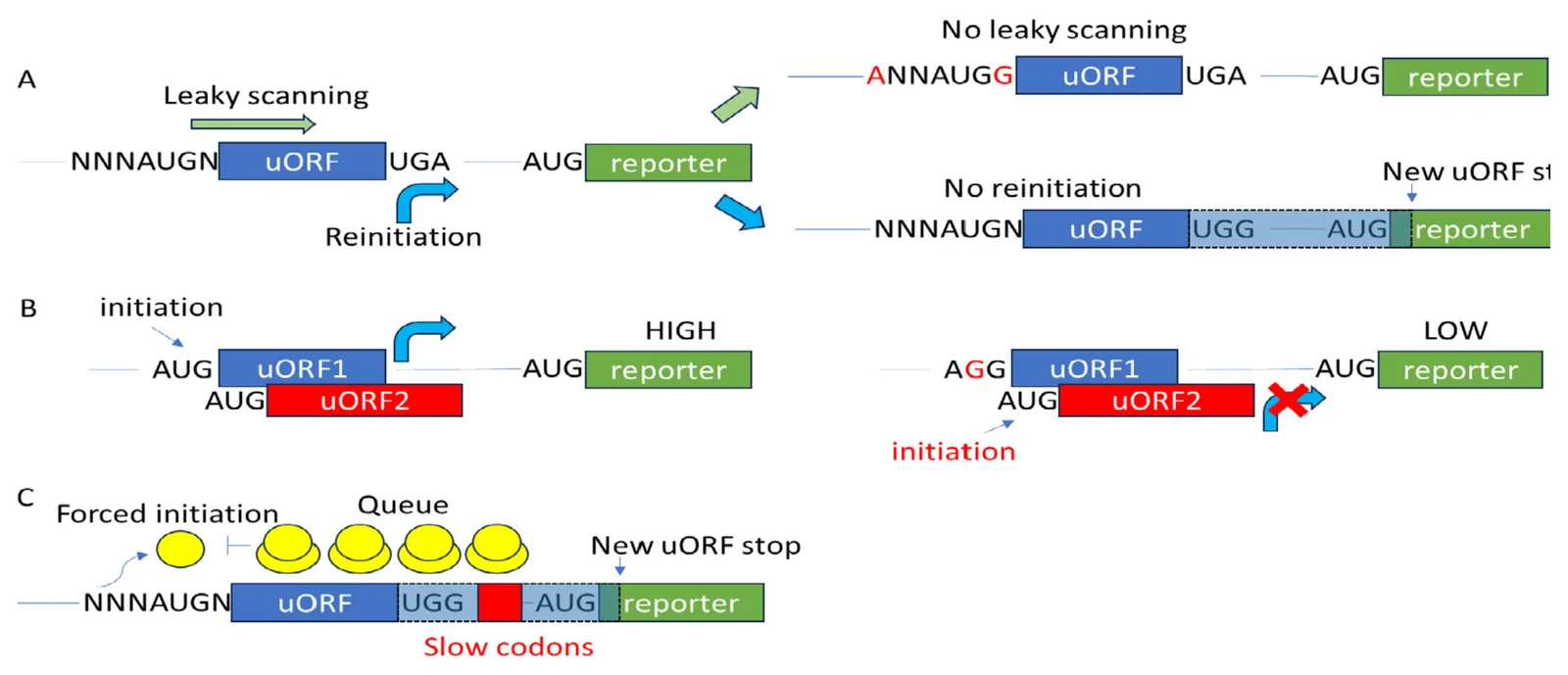
(A) Workflow of uORF reporter analysis to investigate the impact of leaky scanning and reinitiation. (B) Possible confounding results of mutational analysis in the case of a 5′ leader with 2 uORFs. In the wild-type construct, uORF1 is preferentially translated and repressive so that uORF2 is skipped. Removal of uORF1’s AUG codon will lead to uORF2 translation and subsequent reporter repression, (C) Possible effect of extended uORF on uORF translation. uORF stop codon mutation creates a longer uORF sequence which contains non-optimal codons. Slow elongation within this artificial uORF sequence can lead to ribosome stalling and queuing, which in turn can enhance initiation at the uORF start codon.

Our understanding of how uORFs regulate translation mostly originates from experiments utilising reporter constructs.^23–30^ The mutation of the start codon of a uORF to a non-initiating codon allows us to investigate how the uORF affects reporter gene translation, however, it does not allow us to resolve leaky scanning and reinitiation dichotomy. Several manipulations of the uORF’s sequence can help to assess the relative contribution of these two processes to downstream translation (Figure 1A). Improvement of uORF start codon context or introduction of an additional in-frame start codon within the same uORF is expected to decrease leaky scanning. Persistent translation in the presence of such interventions suggests reinitiation. On the other hand, reinitiation can be ruled out by extending uORF beyond the reporter stop codon. Reinitiation upstream of stop codons usually does not occur, thus the residual reporter activity can be attributed to the leaky scanning.

However, experiments with reporter constructs have serious limitations – changing the uORF sequence can affect uORF-mediated mechanisms of translation control. Addressing the effect of an individual uORF on translation can be confounded by potential out-of-frame start codons within uORF (Figure 1B), and it may be difficult to identify them since some may be non-AUG. In this case, the removal of the uORF’s start codon can activate the downstream uORF which is normally not translated. Furthermore, uORF extension by stop codon removal introduces a sequence that is not optimised for translation. This may artificially increase initiation at the uORF start codon due to ribosome queue formation. Such a phenomenon was previously reported for an AUU-initiated uORF in *AZIN1* mRNA, where a ribosome queue enhances initiation at an AUU codon and represses CDS translation.^31^ It is worth noting that in most cases reporters lack native mRNA sequences that may contain cis-acting elements (e.g. those involved in long-range RNA-RNA interactions) that may be important for uORF-mediated translation control. Finally, the RNA levels of the reporters differ from native mRNA levels and this could be important when RNA-binding proteins with limited availability are involved in the regulation.

Reporter constructs have been instrumental in the study of the relative contributions of leaky scanning and reinitiation following uORF translation. However, as previously described, they are not without their limitations that may confound reporter interpretation. Novel approaches for the assessment of the interplay between these mechanisms are required. Ideally, such approaches should enable genome-wide analysis and investigate uORF-mediated regulation in its native context.

## Materials and Methods

TCP-seq and Ribo-seq data utilised in this work were from the Bohlen et al. 2023 dataset^32^ and were accessed via the Trips-Viz transcriptome browser.^33^ The Sequence Read Archive (SRA) run accessions of the samples used are available in supplementary information. The data made available on Trips-Viz are obtained from the SRA and processed in a standardised pipeline. The reads are trimmed of their adapters, and rRNA reads are removed prior to read alignment directly to the Gencode v25 human transcriptome. Ribosome profiling reads are offset on a per-read-length basis to predict the location of the A-site of each ribosome protected fragment. This enables the assignment of each read to one of the three reading frames.

## Results

The variation of ribosome profiling technique called translation complex profiling (TCP-seq) permits the measurement of scanning ribosomes on mRNA.^32,34–38^ This approach is based on the fixation of ribosomal complexes on mRNA by chemical crosslinking followed by RNAse digestion and separation of PIC complexes from translating 80S ribosomes. The information on PIC footprints can be used for both, the assessment of leaky scanning and the scanning of post-terminating ribosomal complexes downstream of uORFs, thus allowing for the assessment of uORF regulatory modality in its native context. Below we show how this could be done using the data obtained by Bohlen et al.^32^ and accessed via the Trips-Viz transcriptome browser.^33^ The Sequence Read Archive (SRA) run accessions of the samples used are available in supplementary information.

**Figure 2:**
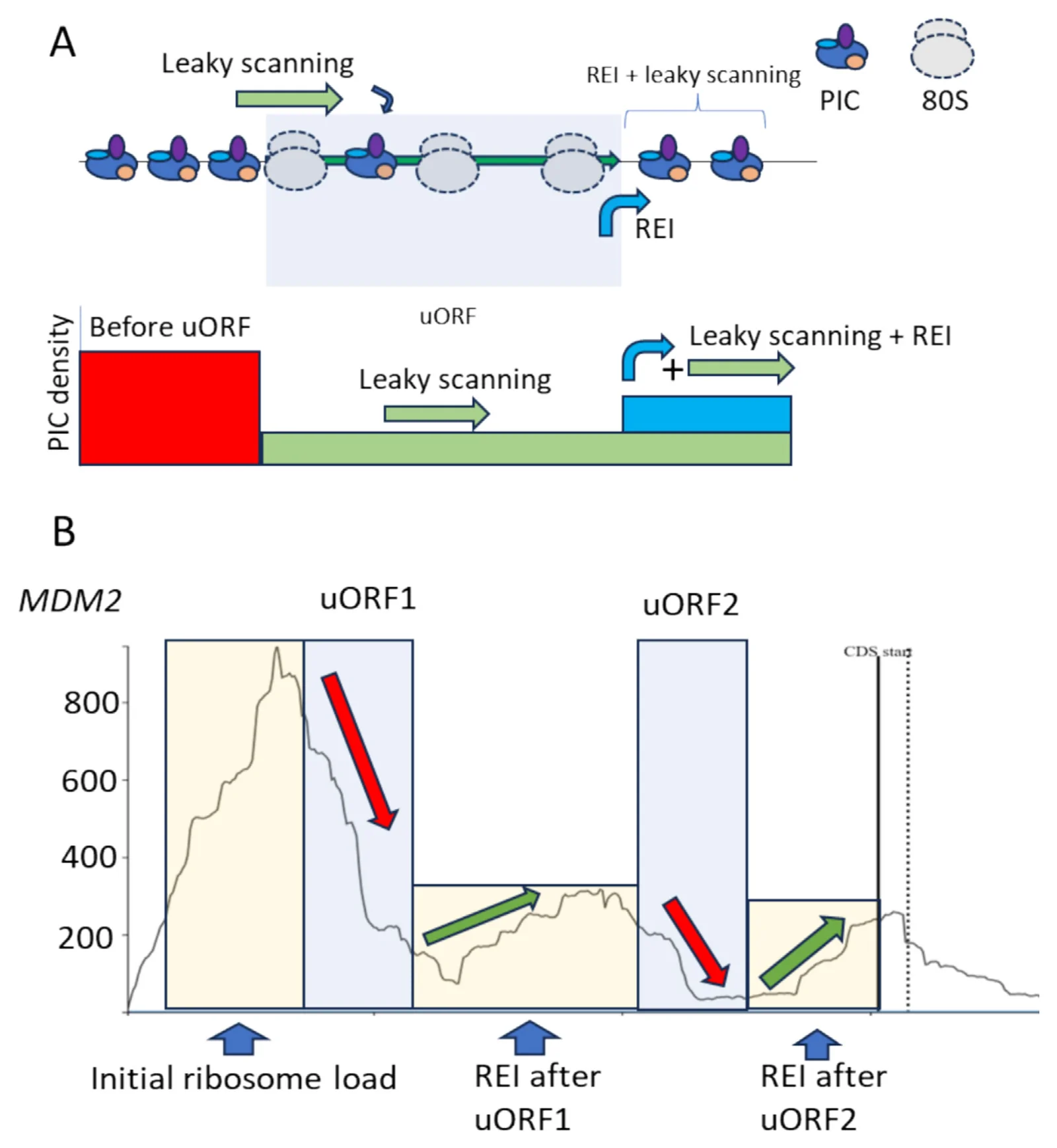
(A) A schematic of expected changes in PIC distribution along 5′ leader with a single uORF. (B) TCP-seq profile (Ribo-seq coverage) of *MDM2* 5′ leader. Red and green arrows show PIC density changes within regions of interest. uORFs are highlighted with blue boxes.

The principle of the approach is shown in Figure 2A. The signal upstream of a uORF is a proxy for PIC loading efficiency onto mRNA 5′ end and scanning towards the uORF start codon (red). PIC density within uORF corresponds to PICs that leaked through uORF start. If no reinitiation occurs downstream of the uORF, the density of PICs would be expected to stay the same, thus an increase in density is likely to occur due to reinitiation (Figure 2A).

We chose *MDM2* as the first example, as *MDM2* contains two uORFs (Figure 2B). Previous experiments with reporter constructs demonstrated that mutation of the uORF1 start codon resulted in a ~4-fold increase in reporter activity while removal of the uORF2 start codon had a modest ~1.5-fold effect.^30^ TCP-seq data show that high PIC signal significantly decreases within the uORF1 and partially restores in the spacer region between uORF1 and uORF2 (Figure 2B). This indicates that uORF1 is indeed repressive, and both leaky scanning and moderate levels of reinitiation contribute to PICs that arrive at the uORF2 start codon. However, a different pattern is observed for uORF2 – PIC density drops to base-line within uORF2 but is almost completely restored after it. Therefore, one can conclude that uORF2 promotes no leaky scanning but it supports very efficient reinitiation.

**Figure 3:**
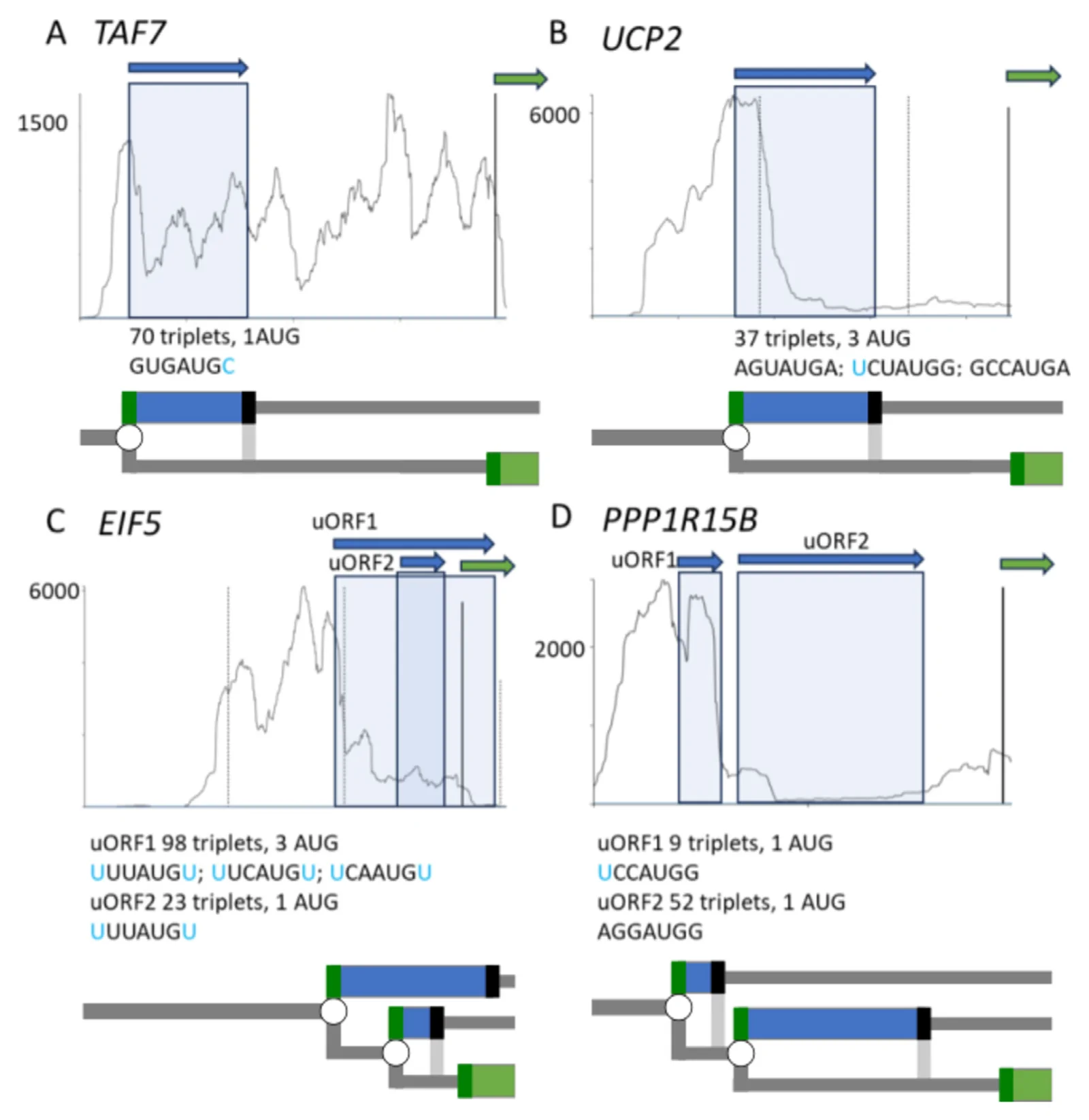
TCP-seq profiles (Ribo-seq coverage) of selected 5′ leaders. uORFs are highlighted with blue boxes and blue arrows, green arrows show the beginning of CDS. The length of each uORF and its AUG start codons are provided below each graph. Nucleotides of non-optimal start codon contexts are shown in blue. Ribosome Decision Graphs^40^ below each panel show the potential ribosome paths that may originate from the translation of the translons annotated with arrows.

To further illustrate the high variability of uORF regulatory modalities we compare TCP-seq profiles of *TAF7* (Figure 3A) and *UCP2* (Figure 3B) mRNA 5′ leaders. *TAF7* contains 70 codons long uORF. The PIC density upstream, downstream and within this uORF is roughly equal which suggests that this uORF is not inhibitory and its start is very leaky. The *UCP2* 5′ leader contains a single uORF which contains three start codons in the optimal nucleotide context. Experiments with reporter constructs demonstrate more than 10-fold inhibitory activity of this uORF.^39^ TCP-seq profile of *UCP2* 5′ leader shows a dramatic gradual reduction of PIC density within the uORF and a slight increase downstream. This is consistent with the expectation that three start codons almost completely preclude leaky scanning and that the uORF does not allow for efficient reinitiation.

Leaky scanning through uORF start codons can operate within regulatory circuits. It was shown that high levels of the initiation factor elF5 induces translation of inhibitory upstream open reading frames (uORFs) in its own mRNA that initiate at AUG codons in conserved poor contexts, and this, in turn, represses elF5 production.^41^ TCP-seq data suggest that PIC density gradually drops ~5-fold inside long uORF1 overlapping CDS consistent with inefficient leaky scanning through uORFs AUG codons (Figure 3C).

A different pattern of PIC distribution is observed for *PPP1R15B* (Figure 3D). Its 5′ leader contains two uORFs. According to experiments with reporter constructs the first shorter uORF1 is not repressive and the second longer uORF2 downregulates translation ~8 fold.^42^ TCP-seq profiling shows that PICs almost completely disappear within uORF2. This indicates that initiation at uORF2 start is very efficient and uORF2 start is not leaky. Instead, PICs reappear after uORF2 which is consistent with reinitiation.

**Figure 4:**
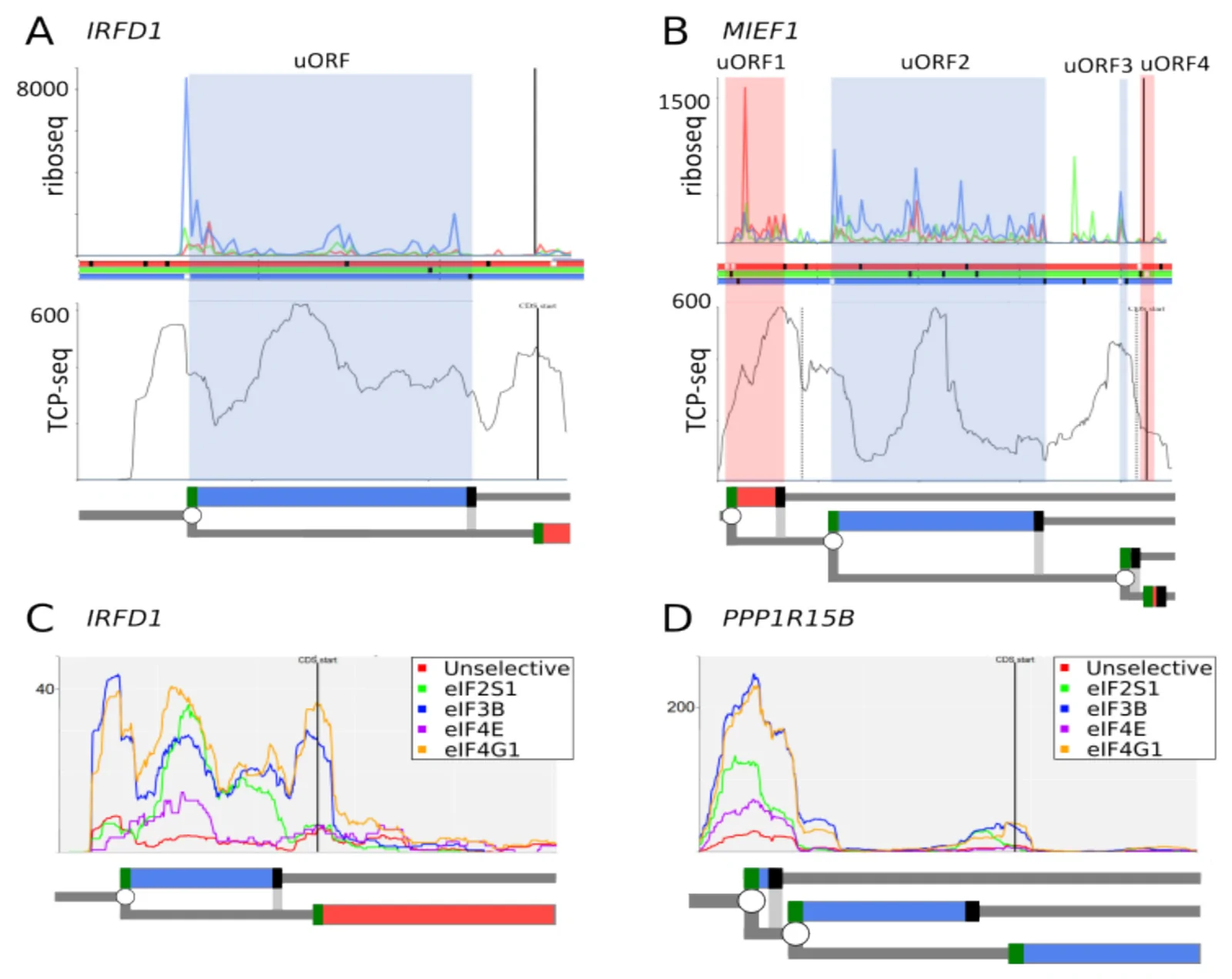
(A and B) Ribo-seq and TCP-seq profiles (Ribo-seq coverage) of *IFRD1* and *MIEF1* 5′ leaders. The top panels are aggregated ribosome profiling data from the Trips-Viz browser. uORFs are highlighted with coloured boxes which correspond to three reading frames (middle panel, white dashes correspond to AUG codons, black dashes – stop codons). Below are Ribosome Decision Graph representations of the depicted loci showing the potential ribosome paths that lead to the data shown in the profiles above. All plots share the same x-axis of coordinates of the transcript isoform. Branch points in the RDG correspond to the AUG codons shown in the ORF plot. C and D: Selective TCP-seq profiles of *PPP1R15B* and *IFRD1* 5′ leaders. Both plots show the same tracks for two different transcripts. Each trace is coloured according to the initiation factor that was selected. Samples aggregated in each trace are recorded in Supplementary Table 1. The read counts represented on the Y axis are normalised by the factor difference in the number of mapped reads between each track akin to the scaling factor used in transcripts per million (TPM) calculations.

In addition to reinitiation and leaky scanning, we also observed unusual patterns in PIC distribution that point to the existence of a novel mode of uORF-mediated translation control. An intriguing example is found for *IFRD1*. This 5′ leader contains a single uORF which represses translation ~8-fold in reporter constructs.^42^ Contrary to the expectation, we found an accumulation of PIC density within the uORF (Figure 4A). Another example was found in the *MIEF1* 5′ leader, where **a peak of PICs density is observed** within the long uORF2 (Figure 4B). We propose that PIC presence within the inhibitory uORF is the result of the queuing of PICs and 80S ribosomes engaged in uORF translation. Probably, PICs do not always dissociate from mRNA upon collisions with 80S but the scanning rate within the uORF is decreased, and this in turn results in translation repression of the main ORF. We hypothesise that such stalled PICs contain additional translation initiation factors which stabilise them on uORF mRNA sequence, and mRNA nucleotides within ribosome’s mRNA tunnel can also stabilise mRNA-ribosome interactions. Such a hypothetical mechanism can be called slow leaky scanning.

Re-emergence of scanning complexes in TCP-seq after the translation of an upstream translon signifies the potential for reinitiation. However, it is the association of initiation factors that dictates the reinitiation ability. The dissociation of the 40S subunit upon termination precludes downstream translation but the continued association of the 40S with the mRNA is not sufficient for reinitiation on its own and the recruitment of initiation factors is still required.

Selective TCP-seq (Sel-TCP-seq), where 40S complexes bound to proteins of interest (initiation factors eIF3B, eIF4E, eIF2S1 and eIF4G1 in the Bohlen dataset) appear to increase the amplitude of the peaks and troughs in the profiles around translons. We posit that this is due to the additional immunoprecipitation step increasing the abundance of bonafide reads from PIC-associated 40S relative to non-PIC originating reads. All initiation factors selected in the Bohlen study, excluding eIF4E, exhibit this increased amplitude downstream of the translon. However, the Sel-TCP-seq profile for eIF2S1-associated 40S complexes differs considerably from the profiles of the structural subunits of the scanning complex that were also profiled (eIF3B and eIF4G1) in some cases. The eIF2 complex plays an essential role in the recruitment of ternary complexes to the P-site during translation initiation making its presence required for reinitiation to occur.

Figure 4B shows two transcripts where the relationship between eIF2S1 and eIF4G1/eIF3B differs. In *PPP1R15B*, eIF3B, eIF4G1 and eIF2S1 re-appear after translation of the upstream translon and this enables the translation of the CDS (as described above). Throughout the translation of the upstream translon in *IFRD1*, eIF3B and eIF4G1 remain associated with scanning complexes at a relatively high rate indicating leaky scanning occurs. Following translation termination of this translon, the abundance of eIF3B and eIF4G1 associated 40S complexes increases while eIF2S1 association significantly decreases. Following the hypothesis that scanning complexes queue across this upstream translon it is plausible that eIF2S1 dissociates due to the increased time spent in the queue.

These examples demonstrate how TCP-seq has the potential to elucidate the leaky scanning/reinitiation dichotomy that remains obfuscated in Ribo-seq data and how further nuances can be obtained via the comparative examination of Sel-TCP-seq profiles. However, the applicability of this approach to a given locus depends on several factors.

To identify reinitiation we look for the following: the presence of scanning complexes 5′ of the translon start, a reduction in scanning complex abundance within the translon, followed by an increased abundance of 40S’s 3′ of the translon stop. As a result, the suitability of this approach for the assessment of reinitiation is dependent on the complexity of the potential signal origins and the translon architecture of the locus. This approach assumes that PICs move along mRNA with roughly uniform velocities. Besides pausing at potential initiation sites, the sequence dependence of the velocity of PICs is currently unknown. Potentially, PIC pausing and/or queueing may result in high density of PIC footprints. This possibility should be taken into account in the interpretation of TCP-seq data using our approach.

As previously mentioned, the population of reads within a TCP-seq sample will consist of both PIC-associated 40S subunits and 40S subunits that were crosslinked to the mRNA while part of an elongating 80S ribosome and subsequently dissociated from the large subunit. As a result, there is the potential for reads from elongating ribosomes that were translating a translon to be misinterpreted as scanning complexes inflating the perceived degree to which leaky scanning occurs. In addition, it should be taken into account that some TCP-seq reads may have a non-ribosomal origin (e.g. from RNA-protein complexes with similar sedimentation properties to that of PIC complexes).

As with any transcriptomic high throughput signal, including Ribo-seq and RNA-seq, the exact transcript isoform from which a given read originated is often impossible to determine in isolation and difficult to infer from a sample. This introduces significant complexities when it comes to extrapolating translation dynamics from aligned ribosome-protected fragments from Ribo-seq and TCP-seq. In the instance where two transcript isoforms of the same gene are expressed to non-aberrant degrees, the signal observed in a profile of one transcript will contain a signal originating from the other as well as from itself on shared exons. Although we are yet to identify a definitive example of this, the re-emergence of the 40S signal downstream of a translon stop may originate from an alternative isoform with a shorter leader sequence.

Short reads are notoriously difficult to map to large genomes/transcriptomes due to the ambiguity introduced by shared tracts of sequence across the reference. TCP-seq datasets have a wider read length distribution than typical Ribo-seq datasets do and as a result, enjoy a greater degree of mappability. However, there can still be issues mapping reads to certain genes and as a result, translons with no read coverage will be observed even in the event of low initiation probabilities. This has the potential to lead to the misattribution of signal to the reinitiation ribosome path rather than leaky scanning.

These aforementioned confounding factors, among others, have the potential to confound the interpretation of translation dynamics using TCP-seq and Ribo-seq. However, existing browsers made available via RiboSeq.org (namely, GWIPs-viz and Trips-Viz) contain much of the necessary data to enable a researcher to identify translation mechanisms despite these factors.

Additional data cannot however increase the number of 5′ leaders with suitable translon architecture to enable the application of this approach. The organisation of translated regions is an important feature of many 5′ leaders that impacts the regulation of downstream CDS translation. However, there exist instances where the organisation of active translons limits the use of this approach. The first instance is that the distance between the stop of translon 1 and the start of translon 2 is too short. In this case, reinitiation may well occur but not be detectable as no reemergence of TCP-seq signal is observed in this region. Furthermore, in cases where there are a large number of active translation initiation sites in one 5′ leader the relative changes in scanning complex abundances may be difficult to delineate, obfuscating the relative contributions of each translon to the change in 40S densities.

Finally, it should be noted that due to the heterogeneity of footprint lengths, it is currently not possible to accurately determine the position of the ribosome A-site within TCP-seq-derived footprints. In order to address this issue, it is essential to investigate the molecular architecture of various PIC subpopulations, which can protect up to 80 nucleotides of mRNA.

## Conclusion

We provide examples of how translation complex profiling (TCP-seq) can help uncover translation regulation at individual uORFs under normal growth conditions due to the availability of the data. Our study suggests that TCP-seq may be instrumental in understanding the mechanistic roles of uORF-mediated regulation under stress conditions or in response to certain metabolites. Furthermore, a number of non-canonical translation initiation factors such as MCTS/DENR heterodimer and eIF2D,^43,44^ eIF4G2,^45–47^ ribosome rescue factor PELOTA,^48^ RNA binding proteins PRRC2A, B, C proteins^49^ and its ortholog Nocte in fruit fly^50^ regulate leaky scanning or reinitiation at certain uORFs. Generation of TCP-seq under knockdown or overexpression conditions for these factors may shed light on how they affect uORF translation globally.

## Supplementary Material

**Appendix A. Supplementary material**

Supplementary material to this article can be found online at https://doi.org/10.1016/j.jmb.2024.168850.

Supplementary Data 1

## Data Availability

All data used in this work are publicly available

## References

[1] Kozak M (1980). Evaluation of the “scanning model” for initiation of protein synthesis in eucaryotes. Cell.

[2] Hinnebusch AG (2017). Structural insights into the mechanism of scanning and start codon recognition in eukaryotic translation initiation. Trends Biochem Sci.

[3] Hinnebusch AG, Ivanov IP, Sonenberg N (2016). Translational control by 5’-untranslated regions of eukaryotic mRNAs. Science.

[4] Shirokikh NE, Preiss T (2018). Translation initiation by cap-dependent ribosome recruitment: Recent insights and open questions. Wiley Interdiscip Rev: RNA.

[5] Hinnebusch AG (2014). The scanning mechanism of eukaryotic translation initiation. Annu Rev Biochem.

[6] Rogozin IB, Kochetov AV, Kondrashov FA, Koonin EV, Milanesi L (2001). Presence of ATG triplets in 5’ untranslated regions of eukaryotic cDNAs correlates with a ‘weak’ context of the start codon. Bioinformatics.

[7] Suzuki Y, Ishihara D, Sasaki M, Nakagawa H, Hata H, Tsunoda T, Watanabe M, Komatsu T, Ota T, Isogai T, Suyama A (2000). Statistical analysis of the 5′ untranslated region of human mRNA using “Oligo-Capped” cDNA libraries. Genomics.

[8] Pesole G, Gissi C, Grillo G, Licciulli F, Liuni S, Saccone C (2000). Analysis of oligonucleotide AUG start codon context in eukariotic mRNAs. Gene.

[9] Davuluri RV, Suzuki Y, Sugano S, Zhang MQ (2000). CART classification of human 5’ UTA sequences. Genome Res.

[10] Calvo SE, Pagliarini DJ, Mootha VK (2009). Upstream open reading frames cause widespread reduction of protein expression and are polymorphic among humans. PNAS.

[11] Dever TE, Ivanov IP, Hinnebusch AG (2023). Translational regulation by uORFs and start codon selection stringency. Genes Dev.

[12] Renz PF, Valdivia-Francia F, Sendoel A (2020). Some like it translated: small ORFs in the 5′UTR. Exp Cell Res.

[13] Silva J, Fernandes R, Romao L (2019). Translational regulation by upstream open reading frames and human diseases. Adv Exp Med Biol.

[14] Chen HH, Tarn WY (2019). uORF-mediated translational control: recently elucidated mechanisms and implications in cancer. RNA Biol.

[15] Zhang H, Wang Y, Lu J (2019). Function and evolution of upstream ORFs in eukaryotes. Trends Biochem Sci.

[16] Wethmar K (2014). The regulatory potential of upstream open reading frames in eukaryotic gene expression. Wiley Interdiscip Rev: RNA.

[17] McGeachy AM, Ingolia NT (2016). Starting too soon: upstream reading frames repress downstream translation. EMBO J.

[18] Johnstone TG, Bazzini AA, Giraldez AJ (2016). Upstream ORFs are prevalent translational repressors in vertebrates. EMBO J.

[19] Ingolia NT, Ghaemmaghami S, Newman JR, Weissman JS (2009). Genome-wide analysis in vivo of translation with nucleotide resolution using ribosome profiling. Science.

[20] Mudge JM, Ruiz-Orera J, Prensner JR, Brunet MA, Calvet F, Jungreis I, Gonzalez JM, Magrane M, Martinez TF, Schulz JF, Yang YT (2022). Standardized annotation of translated open reading frames. Nature Biotechnol.

[21] Kozak M (2001). Constraints on reinitiation of translation in mammals. Nucleic Acids Res.

[22] Mohammad MP, Munzarova Pondelickova V, Zeman J, Gunisova S, Valasek LS (2017). In vivo evidence that eIF3 stays bound to ribosomes elongating and terminating on short upstream ORFs to promote reinitiation. Nucleic Acids Res.

[23] Young SK, Wek RC (2016). Upstream open reading frames differentially regulate gene-specific translation in the integrated stress response. J Biol Chem.

[24] Lee YY, Cevallos RC, Jan E (2009). An upstream open reading frame regulates translation of GADD34 during cellular stresses that induce eIF2alpha phosphorylation. J Biol Chem.

[25] Hanfrey C, Elliott KA, Franceschetti M, Mayer MJ, Illingworth C, Michael AJ (2005). A dual upstream open reading frame-based autoregulatory circuit controlling polyamine-responsive translation. J Biol Chem.

[26] Medenbach J, Seiler M, Hentze MW (2011). Translational control via protein-regulated upstream open reading frames. Cell.

[27] Hardy S, Kostantin E, Wang SJ, Hristova T, Galicia-Vazquez G, Baranov PV, Pelletier J, Tremblay ML (2019). Magnesium-sensitive upstream ORF controls PAL phosphatase expression to mediate energy metabolism. PNAS.

[28] Loughran G, Zhdanov AV, Mikhaylova MS, Rozov FN, Datskevich PN, Kovalchuk SI, Serebryakova MV, Kiniry SJ, Michel AM, O’Connor PBF, Papkovsky DB (2020). Unusually efficient CUG initiation of an overtapping reading frame in POLG mRNA yields novel protein POLGARF. PNAS.

[29] Gunisova S, Valasek LS (2014). Fail-safe mechanism of GCN4 translational control–uORF2 promotes reinitiation by analogous mechanism to uORF1 and thus secures its key role in GCN4 expression. Nucleic Acids Res.

[30] Akulich KA, Sinitcyn PG, Makeeva DS, Andreev DE, Terenin IM, Anisimova AS, Shatsky IN, Dmitriev SE (2019). A novel uORF-based regulatory mechanism controls translation of the human MDM2 and eIF2D mRNAs during stress. Biochimie.

[31] Ivanov IP, Shin BS, Loughran G, Tzani I, Young-Baird SK, Cao C, Atkins JF, Dever TE (2018). Polyamine control of translation elongation regulates start site selection on antizyme inhibitor mRNA via ribosome queuing. Mol Cell.

[32] Bohlen J, Fenzl K, Kramer G, Bukau B, Teleman AA (2020). Selective 40S footprinting reveals cap-tethered ribosome scanning in human cells. Mol Cell.

[33] Kiniry SJ, O’Connor PBF, Michel AM, Baranov PV (2019). Trips-Viz: a transcriptome browser for exploring Ribo-Seq data. Nucleic Acids Res.

[34] Archer SK, Shirokikh NE, Beilharz TH, Preiss T (2016). Dynamics of ribosome scanning and recycling revealed by translation complex profiling. Nature.

[35] Wagner S, Herrmannova A, Hronova V, Gunisova S, Sen ND, Hannan RD, Hinnebusch AG, Shirokikh NE, Preiss T, Valasek LS (2020). Selective translation complex profiling reveals staged initiation and co translational assembly of initiation factor complexes. Mol Cell.

[36] Duncan CDS, Mata J (2022). Translation-complex profiling of fission yeast cells reveals dynamic rearrangements of scanning ribosomal subunits upon nutritional stress. Nucleic Acids Res.

[37] Kito Y, Matsumoto A, Ichihara K, Shiraishi C, Tang R, Hatano A, Matsumoto M, Han P, Iwasaki S, Nakayama KI (2023). The ASC-1 complex promotes translation initiation by scanning ribosomes. EMBO J.

[38] Weiss B, Dikstein R (2024). Unraveling the landscapes and regulation of scanning, leaky scanning, and 48S initiation complex conformations. Cell Rep.

[39] Pecqueur C, Alves-Guerra MC, Gelly C, Levi-Meyrueis C, Couplan E, Collins S, Ricquier D, Bouillaud F, Miroux B (2001). Uncoupling protein 2,in vivo distribution, induction upon oxidative stress, and evidence for translational regulation. J Biol Chem.

[40] Tierney JAS, Swirski M, Tjeldnes H, Mudge JM, Kufel J, Whiffin N, Valen E, Baranov PV (2024). Ribosome decision graphs for the representation of eukaryotic RNA translation complexity. Genome Res.

[41] Loughran G, Sachs MS, Atkins JF, Ivanov IP (2012). Stringency of start codon selection modulates autoregulation of translation initiation factor eIF5. Nucleic Acids Res.

[42] Andreev DE, O’Connor PB, Fahey C, Kenny EM, Terenin IM, Dmitriev SE, Cormican P, Morris DW, Shatsky IN, Baranov PV (2015). Translation of 5’ leaders is pervasive in genes resistant to eIF2 repression. eLife.

[43] Young DJ, Makeeva DS, Zhang F, Anisimova AS, Stolboushkina EA, Ghobakhlou F, Shatsky IN, Dmitriev SE, Hinnebusch AG, Guydosh NR (2018). Tma64/eIF2D, Tma20/MCT-1, and Tma22/DENR recycle post-termination 40S subunits in vivo. Mol Cell.

[44] Schleich S, Strassburger K, Janiesch PC, Koledachkina T, Miller KK, Haneke K, Cheng YS, Kuechler K, Stoecklin G, Duncan KE, Teleman AA (2014). DENR-MCT-1 promotes translation re-initiation downstream of uORFs to control tissue growth. Nature.

[45] Weber R, Kleemann L, Hirschberg I, Chung MY, Valkov E, Igreja C (2022). DAP5 enables main ORF translation on mRNAs with structured and uORF-containing 5’ leaders. Nature Commun.

[46] Smirnova VV, Shestakova ED, Nogina DS, Mishchenko PA, Prikazchikova TA, Zatsepin TS, Kulakovskiy IV, Shatsky IN, Terenin IM (2022). Ribosomal leaky scanning through a translated uORF requires eIF4G2. Nucleic Acids Res.

[47] She R, Luo J, Weissman JS (2023). Translational fidelity screens in mammalian cells reveal eIF3 and eIF4G2 as regulators of start codon selectivity. Nucleic Acids Res.

[48] Fernandez SG, Ferguson L, Ingolia NT (2024). Ribosome rescue factor PELOTA modulates translation start site choice for C/EBPalpha protein isoforrns. Life Sci Alliance.

[49] Bohlen J, Roiuk M, Neff M, Teleman AA (2023). PRRC2 proteins impact translation initiation by promoting leaky scanning. Nucleic Acids Res.

[50] Zhang T, Xue Y, Su S, Altouma V, Ho K, Martindale JL, Lee SK, Shen W, Park A, Zhang Y, De S (2024). RNA-binding protein Nocte regulates Drosophila development by promoting translation reinitiation on mRNAs with long upstream open reading frames. Nucleic Acids Res.

